# Adhesion GPCR GPR56 Expression Profiling in Human Tissues

**DOI:** 10.3390/cells10123557

**Published:** 2021-12-16

**Authors:** Fyn Kaiser, Markus Morawski, Knut Krohn, Nada Rayes, Cheng-Chih Hsiao, Marianne Quaas, Gabriela Aust

**Affiliations:** 1 Research Laboratories and Clinic of Visceral, Transplantation, Thoracic, and Vascular Surgery, Leipzig University and University Hospital Leipzig, 04103 Leipzig, Germany; nada.rayes@medizin.uni-leipzig.de (N.R.); marianne.quaas@medizin.uni-leipzig.de (M.Q.); gabriela.aust@medizin.uni-leipzig.de (G.A.); 2 Paul Flechsig Institute of Brain Research, Leipzig University, 04103 Leipzig, Germany; markus.morawski@medizin.uni-leipzig.de; 3 Core Unit DNA-Technologies, Leipzig University, 04103 Leipzig, Germany; knut.krohn@medizin.uni-leipzig.de; 4 Department of Experimental Immunology, Amsterdam Infection and Immunity Institute, Amsterdam UMC, 1105 Amsterdam, The Netherlands; c.hsiao@amsterdamumc.nl

**Keywords:** GPR56, *ADGRG1*, thyroid, microglia

## Abstract

Despite the immense functional relevance of GPR56 (gene *ADGRG1*) in highly diverse (patho)physiological processes such as tumorigenesis, immune regulation, and brain development, little is known about its exact tissue localization. Here, we validated antibodies for GPR56-specific binding using cells with tagged GPR56 or eliminated *ADGRG1* in immunotechniques. Using the most suitable antibody, we then established the human GPR56 tissue expression profile. Overall, *ADGRG1* RNA-sequencing data of human tissues and GPR56 protein expression correlate very well. In the adult brain especially, microglia are GPR56-positive. Outside the central nervous system, GPR56 is frequently expressed in cuboidal or highly prismatic secreting epithelia. High *ADGRG1* mRNA, present in the thyroid, kidney, and placenta is related to elevated GPR56 in thyrocytes, kidney tubules, and the syncytiotrophoblast, respectively. GPR56 often appears in association with secreted proteins such as pepsinogen A in gastric chief cells and insulin in islet β-cells. In summary, GPR56 shows a broad, not cell-type restricted expression in humans.

## 1. Introduction

GPR56 (gene *ADGRG1*), a member of the adhesion G protein-coupled receptors (GPCRs) superfamily, is involved in a myriad of (patho)physiological processes, such as tumorigenesis, male fertility, immune regulation, and muscle cell hypertrophy [1,2,3,4]. Notably, it has pleiotropic functions in the development and regulation of the central nervous system (CNS) [5,6,7,8,9]. *ADGRG1* loss-of-function mutations cause bilateral frontoparietal polymicrogyria (BFPP), a severe brain malformation [5]. In mice, the receptor regulates oligodendrocyte development [7], CNS myelination [8], and microglial synaptic refinement [9]. Decreased *ADGRG1*/*Adgrg1* levels in the prefrontal cortex are associated with depression in humans and depressive-like behavior in mice [10].

**Figure 1:**
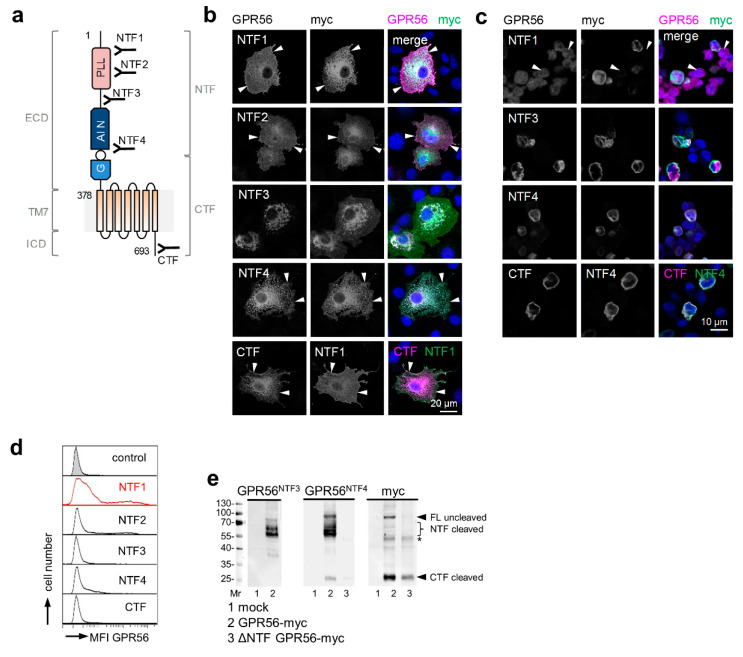
Verification of GPR56 Abs using tagged GPR56. (**a**) Schema of GPR56 and GPR56 Ab binding sites; see running text for explanations. (**b**–**e**) COS-7 cells, transfected with C-terminal myc-tagged or untagged full-length (FL) *ADGRG1* (NM_201524.3), or ΔNTF *ADGRG1* pcDNA3.1 were used to examine GPR56 Ab specificity. (**b**) Monolayered, acetone-fixed GPR56myc cells were costained for GPR56 and myc. All GPR56 Abs bound GPR56 specifically (arrows: membrane-near staining); immunofluorescence. The GPR56^CTF^ Ab binds only the untagged GPR56 C-terminus, therefore, cells transfected with untagged *ADGRG1* were co-stained with the well-established GPR56^NTF1^ Ab. (**c**) GPR56myc cells were detached and paraffin-embedded. Sections were costained with the indicated Abs (see also (**b**)); immunofluorescence. The GPR56^NTF1^ Ab unspecifically stained myc-negative cells (arrows). (**d**) Flow cytometry: GPR56myc Cos-7 cells were analyzed for GPR56 cell surface expression; MFI: mean fluorescence intensity. (**e**) Western blotting: lysates of mock, full-length (FL) GPR56myc or ΔNTF GPR56myc cells were blotted and the indicated Abs applied. The GPR56^NTF4^ Ab bound to uncleaved GPR56 (~86.5 kDa), the cleaved NTF (56.6–60.5 kDa multiple bands), and, inconstantly and weakly, the CTF (~26 kDa). The myc Ab confirmed the results; * likely unspecific bands.

GPR56 shares the typical multimodal structure of adhesion GPCRs (Figure 1a). Its large extracellular domain (ECD), connected to the seven-span transmembrane helices (7TM) and the intracellular domain (ICD), mediates cell–cell and cell–matrix interactions with diverse interaction partners in a cell-type-specific manner [11]. The ECD contains two functional domains: the pentraxin/laminin/neurexin/sex-hormone-binding-globulin-like (PLL) domain and the juxtamembranous GPCR autoproteolysis inducing (GAIN) domain [12]. The latter harbors the GPCR proteolysis site (GPS), where the receptor cleaves into an N- (NTF) and C-terminal fragment (CTF). Due to alternative RNA splicing, GPR56/*ADGRG1* is expected to have at least four protein variants and 17 alternative translation start sites, which have a different, tissue-dependent expression [6,13].

Though GPR56 was first described decades ago [14,15], our knowledge of the tissue profile of GPR56 protein in humans, except for the hematopoietic system [16,17,18], is rather limited. In adults, the thyroid, kidney, placenta, pancreas, and testis showed higher *ADGRG1* levels such as the brain, lung, trachea, colon, small intestine, ovary, and several other tissues [6,15,19]. Only a small number of tissues, like the liver, are *ADGRG1*-negative [6,15,19]. Consequently, tissue RNA-sequencing data predict a broad, non-lineage-specific GPR56 expression [20]. Until now, almost all *ADGRG1* data await confirmation at the protein level. To gain systematic insight, we validated antibodies (Abs) for GPR56-specific binding, including the GPR56^NTF1^ Ab (clone CG-4), which is well established in flow cytometry [3]. We either tagged the protein and demonstrated overlapping of the target with GPR56 Ab labeling or eliminated *ADGRG1* transcripts nearly completely in a cell surface GPR56-positive cell line and compared GPR56 knock-out (KO) clones with the wild type (WT). Immuno-techniques with monolayered or paraffin-embedded cells, as well as screening *ADGRG1*-negative and -positive tissues, led to the selection of Abs used to establish the human GPR56 expression profile.

## 2. Materials and Methods

### 2.1. Ethics Statement

The Ethics Committee of the Medical Faculty of Leipzig University approved the study of human tissues for ADGR expression (no. 028/00, 234/14, 269/17, 386/18, 309/19). All patients gave informed consent. The samples from post mortem brains were provided by the Brain Banking Centre Leipzig of the German Brain Net (GZ 01GI9999-01GI0299), operated by the Paul Flechsig Institute of Brain Research. Case recruitment, acquisition of the patients’ personal data, the protocols, the informed consent forms, performing the autopsy, and handling the autopsy brain material have been ethically approved (no. 282/02, 05/17). Brain tissue processing and analysis are described in detail [21,22].

### 2.2. Antibodies (Abs), Plasmids, and Cell Lines

The Abs used are summarized in Table S1. For clarity, the GPR56 Abs have been indicated together with their binding site (Figure 1a). GPR56 NTF-specific Abs were further numbered starting with NTF1, the one closest to the N-terminus. *ADGRG1* pcDNA3.1 plasmids encode full-length, full-length cleavage-deficient (T383A), or N-terminal truncated (ΔNTF) human GPR56 (NM_201524.3) with or without a C-terminal myc-tag [16]. The plasmids were transfected to suitable host cells using Lipofectamine 2000 (Thermo Fisher Scientific; Darmstadt, Germany) and GPR56 expression was evaluated by various methods. COS-7, EPLC-272H, and BEN cells (DMSZ GmbH, Braunschweig, Germany), as well as Calu-3, H441 (ATCC; LGC Standards GmbH, Wesel, Germany), and KNS-62 cells [23] were cultured in DMEM or RPMI1640, with 10% fetal calf serum.

### 2.3. Generation of GPR56KO Clones

Single guide RNAs (sgRNAs) to *ADGRG1* were designed (www.e-crisp.org/E-CRISP/designcrispr; accessed on 21 February 2018). *ADGRG1* (tccaagagtgactccgtcgg) sgRNA1 and (tccacaccgtggcgttgatc) sgRNA2 targeted exon 3 and 6, respectively. pSpCas9(BB)-2A-GFP (PX458), a gift from Feng Zhang (Addgene plasmid 48138), was digested with BsmB1 and ligated with annealed sgRNAs [24]. BEN cells were transfected with these plasmids and sorted after 24 h for GFP and lacking GPR56 at the cell surface in a FACSAria III (Becton Dickinson, Heidelberg, Germany). Clones of both sgRNA were selected for further studies. gDNA sequencing confirmed mutation of the target loci.

### 2.4. Immunolabeling of Cells and Tissues

#### 2.4.1. Attached Cells and Cryosections

Attached cultured cells or 6 µm tissue cryosections were fixed with 4% paraformaldehyde/PBS or ice-cold acetone for 10 min and immunostained with 2 µg/mL GPR56 Ab (all Abs and reagents for labeling Table S1) at 4 °C overnight. Subsequently, the primary Ab was detected with a fluorophore-labeled secondary Ab at room temperature for 60 min.

For fluorophore-based double immunolabeling, the primary mouse and rabbit Abs were always incubated together, whereas both secondary (anti-mouse and anti-rabbit) Abs were incubated successively. To perform horseradish peroxidase (HRP)-based immunolabeling, the cryosections were treated with 0.3% hydrogen peroxide/PBS for 5 min and incubated afterwards with the primary Ab overnight, followed by a HRP-coupled secondary Ab for 30 min and colorimetric detection using DAB according to the manufacturer protocol. Finally, the sections were counterstained with hematoxylin.

#### 2.4.2. Paraffin-Embedded Cells and Tissues

To validate GPR56 Abs on paraffin-embedded cells, detached cells were either inserted into paraffin in a 1.5 mL tube using the routine embedding protocol, or the cells were injected into an isolated mouse lung, serving as a scaffold, which was paraffin-embedded afterwards. In addition, 6 µm sections of paraffin-embedded cells or tissues were rehydrated. For HRP-based immunolabeling, endogenous peroxidase was blocked with 1.0% hydrogen peroxide/96% ethanol between the steps absolute and 96% ethanol for 5 min.

After antigen retrieval (citrate buffer, pH 6.0, 20 min at 120 °C in a pressure cooker, and cooled down for 25 min), paraffin sections were incubated with 2 µg/mL primary Abs overnight at room temperature. All steps either for HRP- or fluorophore-based immunolabeling followed as described above.

For fluorophore-based colabeling of GPR56 and a lectin in kidney paraffin sections, GPR56 immunostaining was firstly performed as described and afterwards the sections were incubated with the fluorescein-labeled lectin for 1 h at room temperature.

Tissues such as the brain or stomach show high lipofuscin-induced autofluorescence injuring further analysis of fluorophore-stained sections. If required, such tissue sections were pretreated with TrueBlack according to the manufacturer’s instruction to quench auto fluorescence before staining.

In the brain, Ab-binding was visualized with nickel-enhanced avidin-biotin-based immunohistochemistry or fluorophore-based double immunolabeling as described before. For immunohistochemistry, paraffin sections were rehydrated and incubated in 60% methanol/2% hydrogen peroxide for 1 h to block endogenous peroxidase. Sections were pretreated with citrate buffer (pH 6.0, see above) and incubated with PBS/2% BSA/0.3% milk powder/0.5% donkey serum containing 2 µg/mL GPR56 Ab overnight at room temperature. The other steps followed the published protocol [22].

Immunofluorescence stains were imaged by confocal laser scanning microscopy (LSM800 or LSM880 fast Airyscan; Carl Zeiss AG, Oberkochen, Germany). In the case of brightfield microscopy of brain sections, an automated slide scanner microscope was applied (AxioScan Z1; Carl Zeiss).

### 2.5. Flow Cytometry

GPR56 surface expression was quantified by flow cytometry. Cells were incubated with 3 µg/mL primary Ab for 30 min, followed by a fluorophore-labeled secondary Ab for 20 min. Cells were either fixed before or after Ab staining with 1% paraformaldehyde/PBS and analyzed in a FACSAria III.

### 2.6. Western Blot Analysis

COS-7 cells were transfected with the *ADGRG1* plasmids 24 h before lysis. To inhibit N-glycosylation, BEN cells were cultured with 6 µM tunicamycin (Merck, Darmstadt, Germany) for various time lengths. Cells or tissue samples were lysed in M-PER or T-PER lysis buffer, respectively, containing Halt™ protease and phosphatase inhibitor cocktail (Thermo Fisher). Western blot analysis was performed as described [25] using secondary fluorophore-labeled anti-rabbit IRDye 680RD and anti-mouse IRDye 800RD IgG (H + L) Abs (LI-COR Biotechnology, Bad Homburg, Germany). Bands were analyzed with the Odyssey^®^ CLx Imaging System (LI-COR).

### 2.7. qRT-PCR and RNA-Sequencing

Relative *ADGRG1* levels were quantified using the ABsolute qPCR SybrGreen Mix (Thermo Fisher) and primers (forward/reverse) gcggggaggctgagaagagact/caggccagggcagagacgacacag on a Rotor-Gene 3000 thermal cycler (Qiagen, Hilden, Germany) with the ΔCt method [26]. *ADGRG1* was normalized to *RPL27* as an endogenous control.

For RNA-sequencing, 100 ng total RNA was rRNA-depleted using the NEBNext^®^ rRNA Depletion Kit v2 (NEB) and then fragmented. The NEBNext Ultra II Directional RNA Library Prep Kit (NEB) was used for random primed library preparation from this RNA. The barcoded libraries were purified, quantified using the Library Quantification Kit- Illumina/Universal (KAPA Biosystems), and their size distribution was analyzed (FragmentAnalyzer, Agilent). Sequencing of 2 × 150 bp was performed with an Illumina NextSeq 550 sequencer (Illumina, San Diego, CA, USA). After demultiplexing with bcl2fastq software (Illumina, v2.20) and polishing using fastp [27], reads were mapped against the human reference genome (hg38) using HISAT2 [28,29]. Stringtie and the R package Ballgown [30] were employed to calculate differential expression.

## 3. Results

### 3.1. Validating GPR56 Abs with Cells Expressing Tagged GPR56

All GPR56 Abs (Figure 1a) specifically detected the receptor in monolayered GPR56myc COS-7 cells as seen by co-staining of GPR56 and the encoded tag (Figure 1b). Nontransfected cells remained unstained. Cleavage-deficient (T383A) GPR56myc was also detected by the GPR56^NTF4^ Ab (not shown). The GPR56^NTF3^, GPR56^NTF4^, and GPR56^CTF^ Abs also distinguished between GPR56myc- and nontransfected cells after paraffin-embedding (Figure 1c). In flow cytometry, only the GPR56^NTF1^ Ab [3], directed to an epitope near the N-terminus, and the GPR56^NTF2^ Ab bound cell surface GPR56 well (Figure 1d). Both Abs compete with each other (not shown), probably binding to the same epitope, therefore, we only used GPR56^NTF1^ from here on. For Western blot analysis, lysates of COS-7 cells expressing myc-tagged full-length or ΔNTF GPR56 were blotted (Figure 1e). The GPR56^NTF4^ Ab, directed at the GAIN domain, bound to multiple bands at ~56.6–60.5 kDa, to one band at ~86 kDa, and inconstantly and weakly to another band at ~26 kDa. Subsequent blot incubation with the myc-tag Ab confirmed the detection of the GPR56 NTF, probably differently glycosylated, full-length GPR56, and of the GPR56 CTF to a small extent. The GPR56^NTF3^ Ab was little suited to verify the GPR56 NTF molecular weight.

### 3.2. Validating GPR56 Abs Using ADGRG1-Deficient Cells

**Figure 2:**
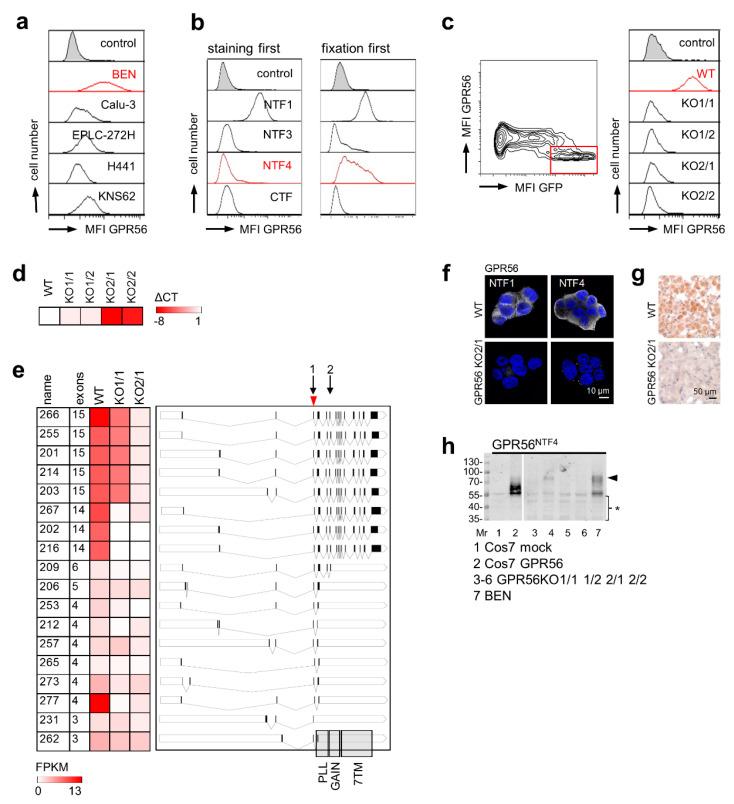
Verification GPR56 Abs comparing BEN cells with *ADGRG1*-deficient BEN clones. (**a**) Human cancer cell lines were analyzed for GPR56 cell surface expression with the GPR56^NTF1^ Ab in flow cytometry. BEN cells, strongly GPR56-positive, were used for generating GPR56 knock-out (KO) clones; MFI, mean fluorescence intensity. (**b**) BEN cells were stained with GPR56 Abs before (left) or after (right) paraformaldehyde-fixation in flow cytometry. (**c**) Selection of BEN cell surface GPR56-negative clones. Left: BEN cells were transfected with *ADGRG1* sgRNA CRISPR/Cas9-GFP plasmid. After 24 h, the cells were stained with the GPR56^NTF1^ Ab, GPR56-negative GFP-positive cells (red bordered) were FACS-sorted and grown as clones. Right: two clones of each *ADGRG1* sgRNA were selected (named KO1/1 etc.), none expressed GPR56 at the cell surface; flow cytometry. (**d**) Quantitation of *ADGRG1* by qRT-PCR. RPL27-normalized log2 x-fold *ADGRG1* mRNA levels of BEN GPR56KO clones compared with BEN WT cells, which were set to 1 (mean, *n* = 3). (**e**) RNA-sequencing data of BEN WT and two GPR56KO clones. Present *ADGRG1* transcripts were named according to the Ensembl database. Left: Abundance of transcripts is given as FPKM (fragments per kilobase per million mapped reads) and visualized by a heat map. Right: Visualization of the present transcript variants. The black boxes represent the transcribed exons. Transcription start sites differ obviously. Binding sites of the *ADGRG1* sgRNA1 (used to generate GPR56KO1 clones) and 2 (GPR56KO2 clones) are indicated by arrows. Arrowhead: translation start site. The grey columns indicate regions where GPR56 domains are encoded. The scheme was generated according to data from Uniprot and Ensembl database (http://wormweb.org/exonintron, accessed on 30 October 2020). (**f**) Monolayered, paraformaldehyde-fixed BEN WT and GPR56KO2/1 cells were stained with the indicated GPR56 Abs; immunofluorescence. GPR56KO2/1 cells are completely GPR56-negative. (**g**) Detached BEN WT and GPR56KO2/1 cells were each injected into an isolated mouse lung (serving as a scaffold) which was paraffin-embedded afterwards. Sections were stained with the GPR56^NTF4^ Ab; immunohistochemistry. (**h**) Western blotting: lysates of mock- and *ADGRG1*-transfected COS-7, BEN WT, and GPR56KO cells were blotted and the GPR56^NTF4^ Ab applied; arrowhead, this specific band also appeared slightly in GPR56KO1/2 cells; * likely unspecific bands.

To find an adherent cell line for knocking out high endogenous *ADGRG1*, we screened carcinoma-derived cell lines using flow cytometry (Figure 2a). BEN cells were strongly GPR56^NTF1^-positive. Remarkably, the receptor was also detectable on the surface with the GPR56^NTF4^ Ab when the cells were fixed first with paraformaldehyde and labeled afterwards (Figure 2b). To eliminate *ADGRG1*, we applied a CRISPR-Cas9-GFP approach and sorted GPR56-negative and GFP-positive BEN cells. All selected clones were GPR56^NTF1^-negative (Figure 2c). *ADGRG1* levels decreased stronger in GPR56KO2 compared to GPR56KO1 clones (Figure 2d). RNA-sequencing confirmed these data: GPR56KO2/1 cells were nearly *ADGRG1*-deficient, whereas in GPR56KO1/2 cells, a few full-length transcripts remained (Figure 2e). In monolayered, paraformaldehyde-fixed BEN cells, the GPR56^NTF1^ and GPR56^NTF4^ Abs labeled intercellular contacts and intracellular structures to a variable extent. GPR56KO2 clones were GPR56-negative with both Abs (Figure 2f). Only the GPR56^NTF4^ Ab also stained GPR56 specifically after paraffin-embedding (Figure 2g). In Western blot analysis, the multiple GPR56-specific bands detected in BEN cells were not present in GPR56KO2 clones. Only in GPR56KO1/2 cells, a faint band remained at ~80 kDa (Figure 2h).

Finally, we selected the GPR56^NTF4^ Ab to localize GPR56 in human tissue. This Ab specifically detected GPR56 on paraformaldehyde-fixed cells in flow cytometry, in paraffin-embedded cells, and Western-blots (Table S2).

### 3.3. GPR56 Is Mainly Present in Non-Squamous Secreting Epithelia

**Figure 3:**
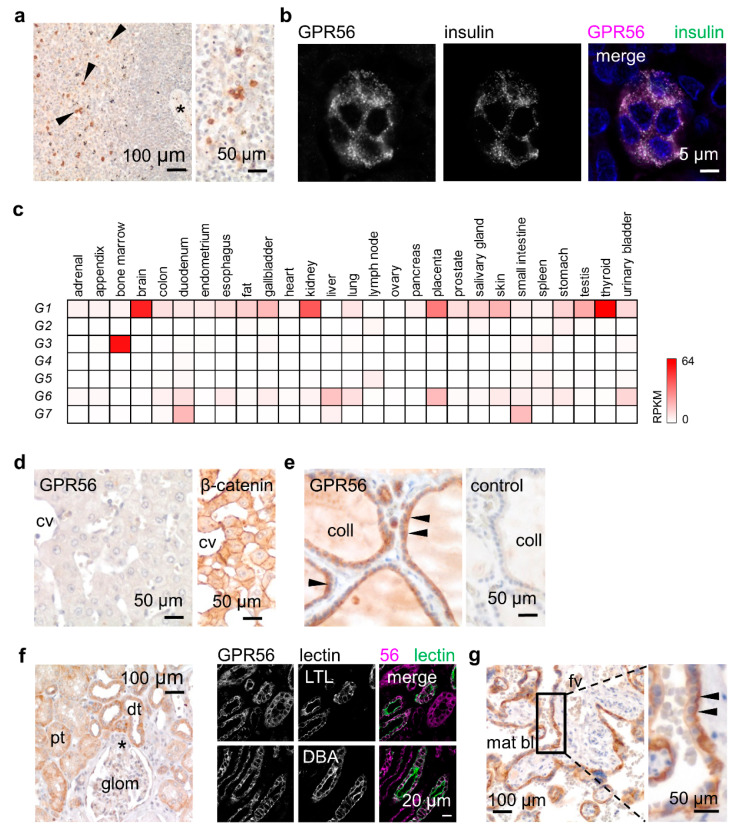
*ADGRG1* and GPR56 expression correlate well in normal human tissues. (**a**,**b**,**d**–**g**) Immunostained paraffin sections using the GPR56^NTF4^ Ab. (**a**) In the spleen, GPR56-positive cells, likely cytotoxic T cells, are located in the mantle zone (arrowheads); *, white pulp artery. (**b**) In pancreatic islets, GPR56 and insulin co-localize in β-cells; double-immunofluorescence. (**c**) Transcriptomic profile of adhesion GPRCs of the G subfamily (*ADGRG1-G7*) in normal human tissues, bulk RNA-sequencing data [20], reads per kilobase exon per million mapped reads (RPKM) are given. (**d**) The liver is GPR56-negative confirming the *ADGRG1* data (**c**). β-catenin was stained as a positive control (cv central vein). (**e**) The thyroid showed the highest *ADGRG1* levels (**c**). Thyrocytes (arrowheads) are strongly GPR56-positive (coll colloid); staining is attenuated at the apical side. (**f**) Kidney tubules express GPR56. Left: pt proximal and dt distal tubules; *, vascular pole of the glom glomerulus. Right: Double-immunofluorescence labeling of GPR56 and tubule-specific lectins. Lotus tetragonolobus lectin (LTL) binds to proximal tubules, *Dolichos biflorus agglutinin* (DBA) to distal tubules. (**g**) In a full-term placenta (mat bl maternal blood, fv fetal vessel), GPR56 appeared attenuated at the basal side of the syncytiotrophoblast (arrowheads).

To verify whether GPR56^NTF4^ staining gives conclusive results, we first screened tissues known to contain GPR56-positive cells. Among circulating leukocytes, only NK and cytotoxic T cells express GPR56 [3,17]. Consequently, only a few lymphocytes of the follicular mantle zone were stained in the spleen (Figure 3a). Furthermore, GPR56 is the most abundant islet-expressed adhesion GPCR [31]. Indeed, in the pancreas, co-staining with insulin confirmed GPR56 in β-cells (Figure 3b).

Afterwards, we analyzed tissues that should contain either none or many GPR56-positive cells, based on bulk tissue RNA-sequencing data (Figure 3c) [20]. Accordingly, the liver, *ADGRG1* negative, remained unstained (Figure 3d). In the thyroid, which shows the highest *ADGRG1* levels, thyrocytes strongly expressed GPR56. Specific staining was partly attenuated at the apical site of these epithelial cells (Figure 3e). RNA data further predict obvious GPR56 in the kidney and placenta. In the kidney, the proximal and distal tubules, identified by co-staining with tubule-specific lectins, expressed GPR56 (Figure 3f). In the mature placenta, the syncytiotrophoblast, the epithelium covering the embryonic placental villi, was strongly GPR56-positive (Figure 3g). In the gastric body, the GPR56-positive cells at the gland base were identified as chief cells by colabeling with pepsinogen A (Figure S1a,b). Pseudostratified tracheal and bronchial epithelia, the serous parts of tracheal and bronchial glands, the epithelia of the prostate and mammary gland, as well as skin sweat glands, all expressed GPR56 at a low to moderate level (Figure S1c–f).

Table S3 summarizes the GPR56 expression profile. Overall, GPR56 protein correlates very well with the *ADGRG1* RNA-sequencing data (Figure 3c) [20]. GPR56 is mainly present in epithelia with isoprismatic/cuboidal cells. Furthermore, some pseudostratified and stratified columnar epithelia expressed GPR56 at a slight to moderate level. In contrast, simple squamous epithelia, such as the endothelium and lung alveolar cells, were GPR56-negative. In stratified squamous epithelia such as the epidermis or the esophageal epithelium, little GPR56 was detectable in the basal and the following suprabasal layers (Figure S1g). With few exceptions, GPR56 was either not present or only present in small amounts in connective tissue like fat, cartilage, and the stroma with fibroblasts, as well as the heart, skeletal and smooth muscle (Figure S1h–k).

Though not shown in detail, the GPR56^NTF4^ Ab stained identical cryo- and paraffin sections of the same tissue. The GPR56^NTF1^ and GPR56^NTF4^ Abs were similarly suitable for immunostaining of cryosections. Consequently, the same cell types were labeled as shown exemplarily for the placenta (Figure S2). The only observed discrepancy persisted between GPR56 detection in smooth muscle cryosections compared with paraffin sections (not shown).

### 3.4. Microglia Express GPR56 in Adult Brains

**Figure 4:**
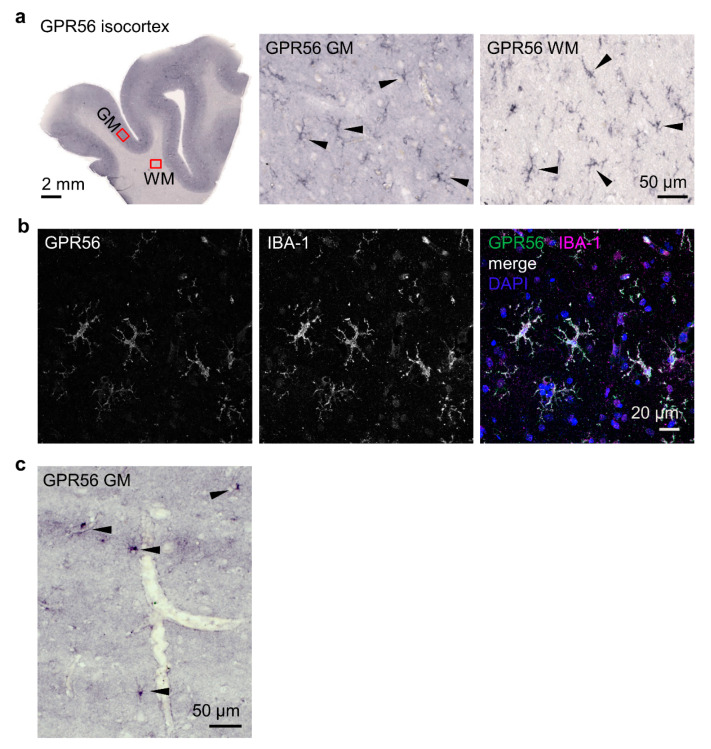
GPR56 is expressed in IBA-1-positive microglia. (**a**–**c**) Immunostained brain paraffin sections using the GPR56^NTF4^ Ab. (**a**) Left: GPR56-immunoreaction in the temporal cortex (A22) grey (GM) and white matter (WM). Middle: GPR56-positive cells, probably microglia cells, in the GM in all layers. Right: GPR56-positive cells, likely microglial cells, in the WM. (**b**) Double immunofluorescence labeling of GPR56 and IBA-1 in the temporal cortex GM. Nuclei were stained with DAPI. All IBA-1-positive microglial cells were GPR56-positive. (**c**) GPR56-positive isolated cells, likely astroglia cells, in the temporal cortex GM.

In contrast to mRNA data, which show high levels of *ADGRG1* in the human brain, the protein has not been localized so far, although research has focused on the extraordinary role of GPR56 in proper cortical development and CNS myelination [5,6,7,8,9,20]. In the adult brain, the GPR56^NTF4^ Ab stained cells, presumably microglia judging by phenotype and localization, in the white and grey matter of isocortical sections of three donors (Figure 4a). Double immunolabeling of GPR56 and ionized calcium-binding adaptor protein-1 (IBA-1), specifically and constitutively expressed in all microglia, confirmed this assumption (Figure 4b). Moreover, astrocytes, localized in the grey matter in all layers, expressed GPR56 (Figure 4c). Overall, GPR56 detection varied strongly among donors. Prolonged time until postmortal brain fixation and/or too long and adverse fixation are likely to have negative effects on epitope availability. The varying quality of various GPR56^NTF4^ Ab batches was further impairing.

### 3.5. Cellular GPR56 Localization Varies Depending on Cell Type and Context

GPR56 is present at the surface of hematopoietic cells such as NK and cytotoxic T cells and of cell lines derived from solid tumors, as shown here (Figure 2a). Interestingly, N-glycosylation facilitates GPR56 surface expression (Figure S3a–c). In BEN cells, after inhibiting N-glycosylation with tunicamycin, the GPR56 molecular weight decreased down to the naked core protein. Various bands with a similar molecular weight appeared, probably representing splice variants. Simultaneously, surface GPR56 decreased, whereas intracellular GPR56 remained unaffected.

In secreting cells, such as thyrocytes and sweat gland cells, GPR56 appeared intracellularly in addition to its slight presence in cell contacts. In islet β- and gastric chief cells, GPR56 co-localized with the secreted proteins, insulin and pepsinogen A, respectively ( Figure 3c and Figure S1b).

**Figure 5:**
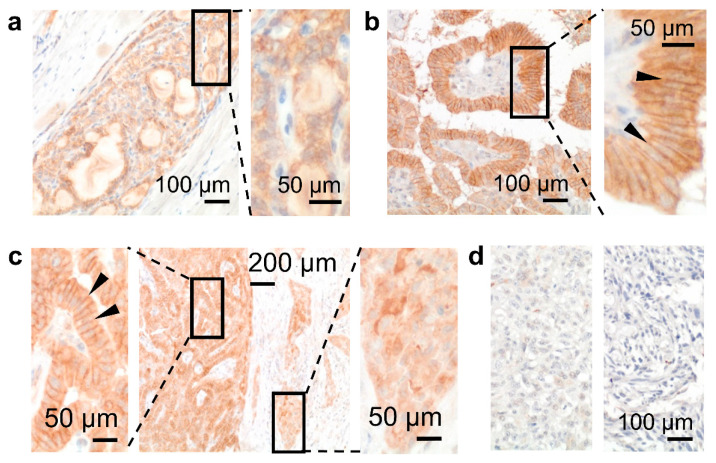
GPR56 localization changed during thyroid tumorigenesis. (**a**) In a follicular carcinoma, GPR56 is located mainly intracellularly as in normal thyrocytes. (**b**,**c**) In papillary carcinomas, GPR56 was strongly upregulated in lateral tumor cell contacts (inserts, arrowheads). Interestingly, at the invasion front GPR56 vanished ((**c**), right insert) and tumor cells located in the invaded capsule showed no membranous staining. (**d**) In a poorly differentiated (left) and in an anaplastic carcinoma (right) GPR56 was strongly downregulated or even disappeared, respectively.

During carcinogenesis, GPR56 cellular localization changed as seen in thyroid tumors (Figure 5a–d, Table S4). GPR56 occurred inside normal thyrocytes (Figure 3e); this pattern mainly remained in a follicular carcinoma (Figure 5a). In differentiated papillary carcinomas (*n* = 7), GPR56 increased strongly in lateral cell-cell contacts (Figure 5b) but vanished from them in tumor cells at the invasion front (Figure 5c). Consequently, GPR56 was only slightly present in an aggressive, poorly differentiated tumor, or even vanished from an anaplastic carcinoma (Figure 5d).

In summary, cellular GPR56 localization varied, obviously indicating different cell type and context-specific functions.

## 4. Discussion

To bridge the gap in our knowledge of GPR56 expression in humans, we profiled this receptor with Abs validated on cells with tagged or eliminated GPR56. The selected GPR56^NTF4^ Ab, suitable for cryo- and paraffin sections, most likely binds to a linear, difficult to access epitope, where a stretch of contiguous amino acids is sufficient for binding. The GPR56^NTF1^ Ab, staining cryosections only, probably detects a conformational epitope where the proximity of key amino acids is lost during paraffin embedding.

Ultimately, the high genomic, structural, and cellular localization and ligand binding variability of GPR56 motivated us to carry out the careful Ab validation. A broad *ADGRG1* transcript repertoire exists, caused by multiple exon combinations and intra-gene transcription start sites. In BEN cells, which show the third-highest *ADGRG1* level within the cancer cell line encyclopedia (portals.broadinstitute.org/ccle), we found >20 Ensembl-annotated *ADGRG1* transcripts. They were absent in the generated GPR56KO2/1 BEN cells, which are, thus, a very good negative control to validate GPR56 Abs. We also found a high variability upstream of the transcription start site in exon 3 in BEN cells. Consequently, analysis of deeply sequenced transcriptomes of three mouse tissues yielded 67 *Adgrg1* transcript variants [32]. In these transcriptomes, 45 transcription start sites in 16 different 5′ start exons were found, indicating multiple promoters, many of them likely to be tissue-specific. Additionally, the *ADGRG1* coding region undergoes alternative splicing to generate a range of variants. In the *ADGRG1* variant S4, found by database search [13], an alternative start codon ATG in exon 4 is initiated, resulting in a variant without the extracellular PLL domain. Mice expressing this variant were first described as *Adgrg1^−/−^* mice [33]. As shown recently, the S4 variant is required for microglia-mediated synaptic pruning [9].

GPR56 variability is further enhanced through glycosylation. In lysates of GPR56myc COS-7, we found multiple bands in Western blots using the GPR56^NTF4^ Ab. Interestingly, N-glycosylation facilitates GPR56 cell surface expression. Monolayered HEK293T cells transfected with N-glycosylation mutant *ADGRG1* showed intracellular, but no longer membrane-associated, GPR56 [34]. Consequently, tunicamycin, which inhibits N-glycosylation, decreased cell surface but not intracellular GPR56 in BEN cells.

GPR56 binds to diverse endogenous extracellular matrix and soluble ligands. Tissue transglutaminase 2, collagen III (α1), and heparin, the growth factor progastrin, all interact with the GPR56 NTF [1,35,36,37]. The diversity of GPR56 ligands is likely to be much higher. Especially intracellularly localized GPR56 and GPR56 in lateral cell–cell contacts as seen in the present study in islet β- and gastric chief cells, thyrocytes, and differentiated thyroid carcinomas, will be part of yet unknown protein complexes. High-resolution microscopy shows GPR56 to be strongly co-localized with the synthesized peptides in islet β- and gastric chief cells. However, fixation, embedding, and antigen retrieval result in various degrees of substance extraction, loss of matrix material, and protein misfolding, all affecting protein co-localization. Thus, the direct interaction between GPR56 and the secreted proteins must be confirmed.

GPR56 shows a prominent epithelial expression profile. We found GPR56 in many peptide/protein-producing cells such as the thyroid epithelial, gastric chief, syncytiotrophoblast, and islet β-cells. Furthermore, the skin sweat and sebaceous glands, the serous part of bronchial and tracheal glands, the distal and proximal kidney tubules, and the pancreatic duct epithelium all consist of cells that mainly absorb and/or secrete electrolyte fluids. Thyrocytes produce the iodoglycoprotein thyroglobulin, stored in the follicular lumen, and synthesize the hormones triiodothyronine (T3) and thyroxine (T4). In the stomach, GPR56 is mainly found in pepsinogen A-positive cells. Pepsinogen is a powerful and abundant digestive enzyme secreted by the chief cells as a proenzyme and then converted to the active enzyme pepsin by gastric acid in the lumen. The placental syncytiotrophoblast synthesizes the peptide hormone human chorionic gonadotropin (hCG), the key embryonic signal essential for the maintenance of pregnancy. Furthermore, we confirmed the known presence of GPR56 in pancreatic islet β-cells [31]. The function of GPR56 in insulin secretion has been recently examined using a mouse expressing the *Adgrg1* S4 variant, which lacks the extracellular PLL domain [31]. This S4 variant did not affect glucose-induced insulin secretion in vitro or impair glucose tolerance in adult mice. However, glucose-induced insulin secretion was potentiated from wild-type islets by the GPR56 ligand collagen III [35]. This effect was absent in *Adgrg1* S4 islets.

Investigating the role of GPR56 during epithelial tumor progression, local invasion, and metastasis formation will be worthwhile, as seen in a small cohort of thyroid cancers. GPR56 upregulation at lateral tumor cell contacts in well-differentiated papillary carcinomas was paralleled by its disappearance, therefrom, in tumor cells at the invasion front. In most aggressive, poorly differentiated, and worse anaplastic carcinomas, GPR56 decreased or even vanished. Thus, epithelial-mesenchymal transition (EMT) or EMT-like dedifferentiation is characterized by decreased GPR56 as known from the loss of E-cadherin and membranous β-catenin (*CTNNB1*) [38].

GPR56 detection remains challenging, as experienced in human brain samples where labeling varied depending on the donor and quality of the GPR56^NTF4^ Ab. In the adult isocortex, GPR56 appeared in IBA-1-positive microglia, the brain-resident phagocytes, and to a lesser extent in astrocytes. That confirms detailed bulk and single-cell RNA-sequencing data in humans characterizing *ADGRG1* as a homeostatic microglia marker [39] and in mice, where *Adgrg1* shows an isolated microglial expression [40,41]. *ADGRG1* is also present at a much lower level in other cell types of the central nervous system. GPR56 was detected at acutely isolated white matter microglia when applying the GPR56^NTF1^ Ab in flow cytometry, whereas choroid plexus macrophages were GPR56-negative in line with the known absence of *ADGRG1* in myeloid cells other than microglia [42]. It is very likely that GPR56 localization differs between fetal and adult human brains. In the fetal brain, GPR56 colocalizes with the transcription factor RFX1 in germinal zones [6].

In summary, GPR56 tissue profiling based on immunotechniques with validated Abs will allow the understanding of how this adhesion GPCR contributes to yet unknown (patho)physiological processes, especially in normal and malignant epithelia.

## Data Availability

The data presented in this study are included in this published article, or are available from the corresponding author upon reasonable request.

## Supplementary Materials

The following are available online at https://www.mdpi.com/article/10.3390/cells10123557/s1, Figure S1: Secreting epithelia express GPR56; Figure S2: The GPR56^NTF4^ Ab stained cryo- and paraffin sections comparably; Figure S3: GPR56 cell surface expression depends on N-glycosylation. Table S1: Antibodies (Abs) and reagents for immunolabeling; Table S2: GPR56 Ab validation; Table S3: GPR56 human expression profile; Table S4: GPR56 in thyroid cancer.

## Abbreviations

adhesion GPCR	adhesion G-protein-coupled receptor
Ab	antibody
CNS	central nervous system
CTF	C-terminal fragment
ECD	extracellular domain
GPCR	G-protein-coupled receptor
GAIN	GPCR autoproteolysis-inducing
GPS	GPCR proteolysis site
ICD	intracellular domain
KO	knock-out
NTF	N-terminal fragment
WT	wild type
