# Supplementary material for: Adhesion GPCR GPR56 Expression Profiling in Human Tissues
Source: Cells. 2021 Dec 16;10(12):3557. doi: 10.3390/cells10123557 (PMC8700376; doi:10.3390/cells10123557)
Supplement: Supplementary file 1 [file cells-10-03557-s001.zip › cells-1488946-supplementary.pdf]

Supplementary information

Adhesion GPCR GPR56 expression profiling in human tissues

Fyn Kaiser [1]*, Markus Morawski [2], Knut Krohn [3], Nada Rayes [1], Cheng-Chih Hsiao [4], Marianne Quaas [1], Gabriela Aust [1]

[1] Research Laboratories and Clinic of Visceral, Transplantation, Thoracic, and Vascular Surgery, Leipzig University, and University Hospital Leipzig; fyn.kaiser@medizin.uni-leipzig.de, nada.rayes@medizin.uni-leipzig.de, marianne.quaas@medizin.uni-leipzig.de, gabriela.aust@medizin.uni-leipzig.de

[2] Paul Flechsig Institute of Brain Research, Leipzig University; markus.morawski@medizin.uni-leipzig.de

[2] Core Unit DNA-Technologies, Leipzig University, Leipzig, Germany; knut.krohn@medizin.uni-leipzig.de

[2] Department of Experimental Immunology, Amsterdam Infection and Immunity Institute, Amsterdam UMC, Amsterdam, Netherlands; c.hsiao@amsterdamumc.nl

* Correspondence: fyn.kaiser@medizin.uni-leipzig.de; Tel.: 0049 341 97 17555

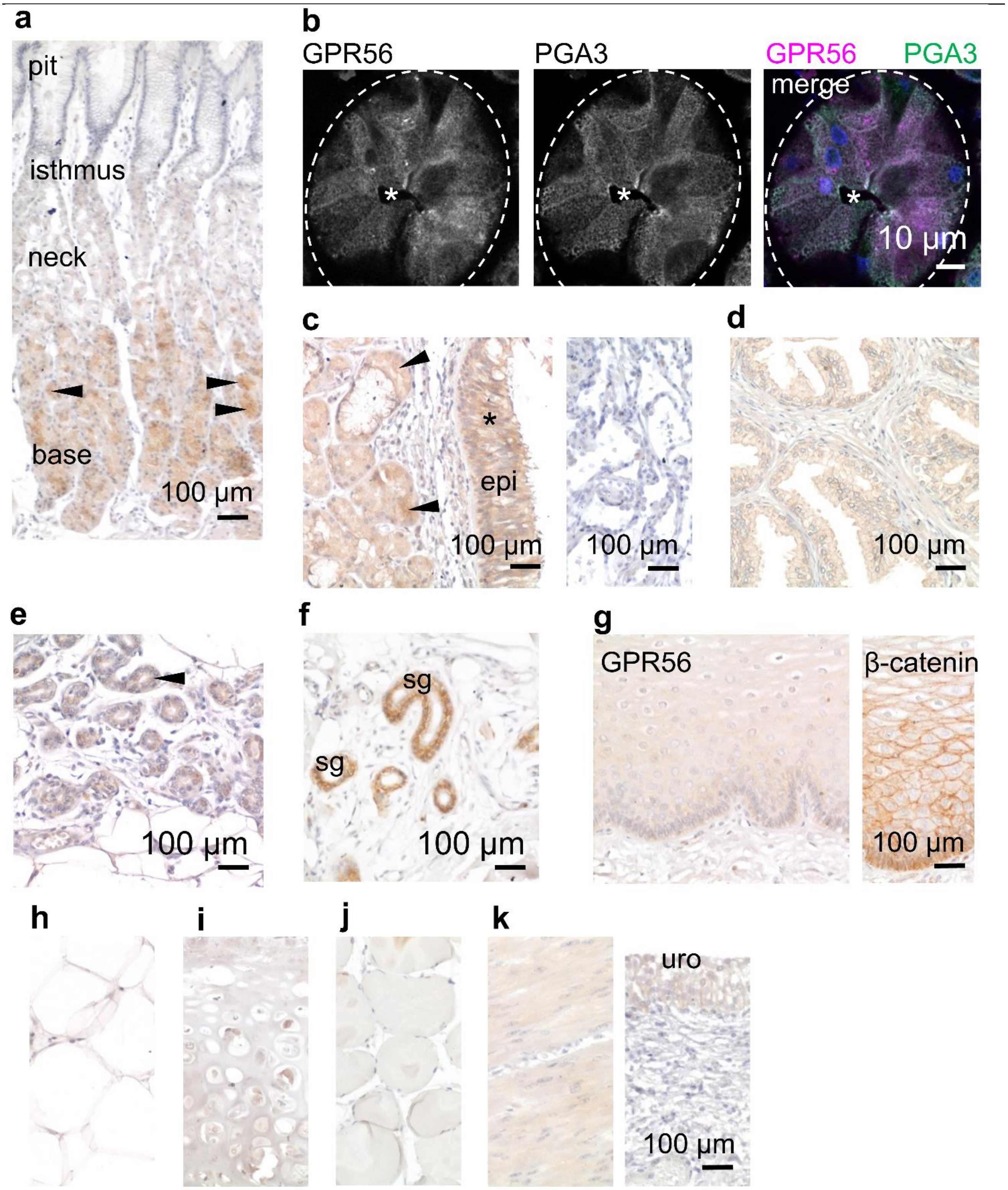

Figure S1 Secreting epithelia express GPR56

(a-k) Immunostained human paraffin sections using the GPR56^{NTF4} Ab. **(a)** In a longitudinal section through the gastric epithelium (corpus region), cells at the gland base express GPR56 (arrowheads). **(b)** GPR56 and pepsinogen A (PGA3) co-localize in chief cells at the gastric gland base (cross section; asterisk: gland lumen), double-immunofluorescence. **(c)** In the lung, the pseudostratified epithelium (asterisk) of an intrapulmonary bronchus and the serous parts of bronchial glands (arrowheads) express GPR56 (left), whereas the alveolar epithelium and thus alveolar type 1 (AT1) and 2 (AT2) cells were GPR56-negative (right). **(d-f)** Epithelial cells in the prostate (**d**), the adult mammary gland (**e**, arrowhead: non-lactating acini), and the sweat glands (sg) of the skin (**f**) are GPR56-positive. (**g**) In the esophagus, basal and the following suprabasal

epithelial cells are hardly GPR56-positive (left GPR56, right β-catenin/*CTNNB1*) **(h-k)** Connective tissue as white fat (**h**) and hyaline cartilage (**i**, trachea), as well as skeletal (**j**, cross section) and smooth muscle (**k**, urinary bladder; left smooth muscle, right uro urothelium) do not or only slightly express GPR56.

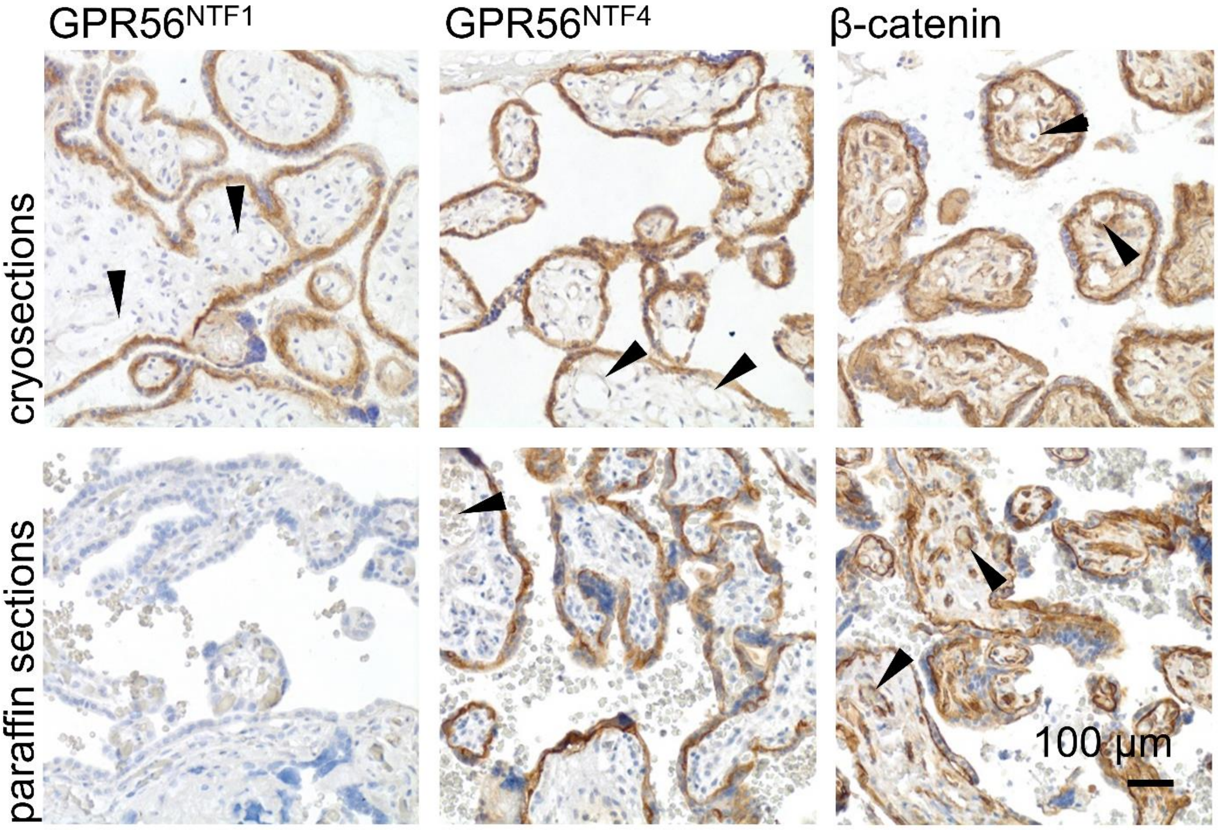

Figure S2 The GPR56^{NTF4} Ab stained cryo- and paraffin sections comparably.

Sections of a full-term placenta were stained with the GPR56^{NTF1} and GPR56^{NTF4} Abs. GPR56 localized in the syncytiotrophoblast, but not, as β-catenin/*CTNNB1* (right, positive control), in the endothelium (arrows).

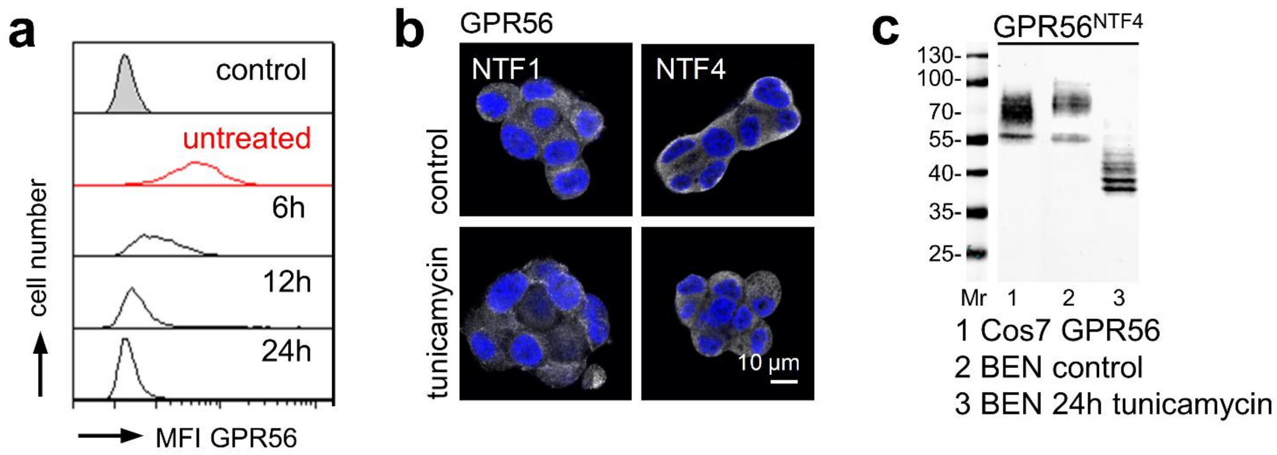

Figure S3 GPR56 cell surface expression depends on N-glycosylation.

(a-c) Tunicamycin, which prevents N-glycosylation, decreased cell surface but not intracellular GPR56. Loss of surface GPR56 was paralleled by a decrease of the GPR56 molecular weight. **(a)** BEN cells, treated with tunicamycin for the indicated times, were stained with the GPR56^{NTF1} Ab and analyzed by flow cytometry; MFI (mean fluorescence intensity). **(b)** Monolayered BEN cells, treated with tunicamycin for 24 h, were stained for GPR56. **(c)** Western blot analysis of GPR56 COS-7 cells and BEN control and tunicamycin-treated cells using the GPR56^{NTF4} Ab. The various bands seen after tunicamycin treatment (line 3) are likely to represent *ADGRG1* splice variants.

Tab. S1. Antibodies (Abs) and reagents for immunolabeling

**Ab/reagent**	**Antigen/binding to**	source, mono/poly clonal	clone	Used for	reference/ company; catalog number
GPR56^{NTF1} Ab	GPR56 nearby N-terminus	m, mono	CG-4	GPR56 Ab screening	[3]
GPR56^{NTF2} Ab	GPR56, competes GPR56^{NTF1} *vice versa*	h, mono	REA467	GPR56 Ab screening	Miltenyi Biotech, Bergisch-Gladbach, Germany; 130-106-979
GPR56^{NTF3} Ab	GPR56, H252-Y265*	r, poly		GPR56 Ab screening	Thermo Fisher Scientific, Darmstadt, Germany; 720373
GPR56^{NTF4} Ab #	GPR56, L289-H381*, GAIN	m, mono	G-6	GPR56 Ab screening	Santa Cruz Biotechnology, Heidelberg, Germany; sc-390192
GPR56^{CTF} Ab	GPR56, C-terminus	r, poly		GPR56 Ab screening	Merck, Darmstadt, Germany; ABS1028
β-catenin Ab		m, mono	C-14	Comparison to GPR56 in tissues	BD Biosciences, Heidelberg, Germany; 610153
β-actin Ab		m, mono	AC-15	Loading control Western blotting	Merck; A5441
myc-Tag Ab		m, mono	9B11	Coimunolabeling using tagged GPR56	Cell Signaling Tech., Danvers, US; 2276
myc-Tag Ab		r, mono	71D10	Coimunolabeling using tagged GPR56	Cell Signaling Tech; 2278
Insulin		gp, p		Coimunolabeling with pancreas	Dako Agilent , Santa Clara, CA, US; #A0564
Pepsinogen A1		r, poly		Coimunolabeling with stomach	Thermo Fisher; PA561360
IBA-1 Ab		r, poly		Coimunolabeling with brain	FUJIFILM Wako Chemicals, Richmond, USA; 019-19741
Alexa Fluor488 anti-rabbit IgG	anti-rabbit	g, $F(ab')_2$ fragment		Secondary Ab fluorophor-based immunolabeling	Jackson ImmunoResearch Europe Ltd, Cambridgeshire, UK; 111-546-144
Alexa Fluor546 anti-mouse IgG	anti-mouse	g, $F(ab')_2$ fragment		Secondary Ab fluorophor-based immunolabeling	Thermo Fisher; A-11018
Lotus tetragonolobus lect in (LTL),	α-linked L-fucose containing oligosaccharides			Colabeling kidney	Vector Laboratories, Burlingame, California, USA; FL-1321

fluorescein-labelled					
Dolichos biflorus agglutinin (DBA), fluorescein-labelled	N-Acetylgalactosamine			Colabeling kidney	Vector Laboratories; FL-1031
ImmPress HRP	anti-mouse	ho, polymer IgG		HRP-based immunolabeling	Vector Laboratories; MP-7402
SIGMAFAST™ DAB tablets				colorimetric detection HRP	Merck; D4418
TrueBlack®				auto fluorescence quencher	Biotium Inc., Fremont, California, USA; #23007

* NP_001139243.1; d donkey, g goat, gp guinea pig, h human, ho horse, m mouse, r rabbit; # GPR56^{NTF4} Ab quality varied obviously between various batches; however, the company did not improve Ab quality until now.

Tab. S2 GPR56 Abs validation

		**GPR56 Ab**			
**method**	**cells/tissues**	$GPR56^{NTF1}$	$GPR56^{NTF3}$	$GPR56^{NTF4}$	$GPR56^{CTF}$
IS	GPR56 COS-7	specific	specific #	specific	specific
IS-P	GPR56 COS-7	-	specific #	specific	specific
IS*	BEN, GPR56KO	specific	-	specific	-
IS-P	BEN, GPR56KO	-	-	specific	-
IH**-cryosection	thyroid	specific	-	specific	-
IH-P	thyroid	-	specific #	specific	-
FC	GPR56 COS-7;<br>BEN, GPR56KO	specific	-	-	-
FC*	GPR56 COS-7;<br>BEN, GPR56KO	specific	-	specific	-
Western Blot	hGPR56 COS-7;<br>BEN, GPR56KO	-	specific	specific	-

FC flow cytometry, IH immunohistology, IS immunostaining of cells; P paraffin-embedded; *paraformaldehyde-fixed; **acetone-fixed; - negative, not suitable, or not clear without ambiguity; # $GPR56^{NTF3}$ stains nuclei of all (also non-transfected) COS-7 cells. This is likely unspecific; nuclear localization sequence prediction using cNLS Mapper resulted in low scores (2-3) for GPR56.

Tab. S3 GPR56 human expression profile (without nervous system)

**Organ System - Cell Type**	**GPR56^{NTF4} Ab staining intensity, pattern**
**hematopoietic**	
Spleen - lymphocytes	few cells mantle zone +++, membranous
**Cardiovascular**	
Heart myocytes	- / ±
Vasculature - endothelial cells, smooth muscle cells, pericytes	-
**Respiratory system**	
Lung – bronchus- epithelial cells	+
Lung - glandulae bronchiales	+ serous parts, cell contacts
Lung - alveolar cells	-
**Digestive system**	
Esophagus- epithelium	± , cell contacts
Stomach - epithelium- mucous (neck) cells, parietal, enteroendocrine cells	- / ±
Stomach - epithelium - chief cells	++, intracellular
Small intestine, colon - epithelium	- / ±
Liver - hepatocytes	-
Pancreas - exocrine, interlobular duct epithelial cells	± / +
**Urinary system**	
Kidney – tubules - proximal, distal	+++, +++, cell contacts
Urinary bladder - urothelium	±
Urinary bladder - smooth muscle	- / ±
**Endocrine System**	
Thyroid - thyrocytes	++/+++, mainly intracellular
Thyroid - C cells	-
Pancreas - endocrine cells (islets of Langerhans)	+++ only β-cells, intracellular
Intestine- enteroendocrine cells	-
**Reproductive system**	
Prostate - epithelial cells	+
Placenta - syncytiotrophoblast	+++, cell contacts, intracellular
**Skin**	
Skin – epidermis - keratinocytes	± basal, most suprabasal layers
Skin - sweat and sebaceous glands (epithelial cells)	++, cell contacts

Breast - mammary gland epithelial cells	± /+
**Musculoskeletal, connective tissue**	
Adipocytes	-
Fibroblasts	-
Cartilage - chondrocytes	-
Skeletal muscle – myocytes	- / ±

immunohistology, paraffin-embedded (IH-P);
–, ± /+ , ++ and +++ indicate no, slight, moderate, and strong staining, respectively

Tab. S4 GPR56 in thyroid cancer

		tumor center			tumor invasion front		normal tissue*	
	type	localization	intensity	GPR56+ (%)	localization	intensity	localization	intensity
1	papillary	m, c	++/+++	100	c	+/++	c	+/++
2	papillary	m, c	+	100			c	+
3	papillary	m, c	++/+++	100			c	+/++
4	papillary	c (m)	+ - +++	100			c	+/++
5	papillary	m, c	++/+++	100	c	+	c	+/++
6	papillary	c (m)	+- +++	100	c	+/++	c	+
7	follicular	c	+/++	90	c	++	c	+/++
8	poorly differentiated	c	±	90				
9	anaplastic	-	-	-				

IH-P immunohistology, paraffin-embedded;
−, ± /+, ++ and +++ indicate no, slight, moderate, and strong staining, respectively;
c cytoplasmatic, m membranous; * if present in the same section
